# The effects of sulfated secondary bile acids on intestinal barrier function and immune response in an inflammatory *in vitro* human intestinal model

**DOI:** 10.1016/j.heliyon.2022.e08883

**Published:** 2022-02-02

**Authors:** Benthe van der Lugt, Maartje C.P. Vos, Mechteld Grootte Bromhaar, Noortje Ijssennagger, Frank Vrieling, Jocelijn Meijerink, Wilma T. Steegenga

**Affiliations:** a Division of Human Nutrition and Health, Wageningen University & Research, Wageningen, the Netherlands; b Department of Molecular Cancer Research, Center for Molecular Medicine, University Medical Center Utrecht, the Netherlands

**Keywords:** Inflammatory bowel disease, Secondary bile acids, Sulfation, Intestinal barrier function, Mucus layer

## Abstract

Dysbiosis-related perturbations in bile acid (BA) metabolism were observed in inflammatory bowel disease (IBD) patients, which was characterized by increased levels of sulfated BAs at the expense of secondary BAs. However, the exact effects of sulfated BAs on the etiology of IBD are not investigated yet. Therefore, we aimed to investigate the effects of sulfated deoxycholic acid (DCA), sulfated lithocholic acid (LCA) and their unsulfated forms on intestinal barrier function and immune response. To this end, we first established a novel *in vitro* human intestinal model to mimic chronic intestinal inflammation as seen during IBD. This model consisted of a co-culture of Caco-2 and HT29-MTX-E12 cells grown on a semi-wet interface with mechanical stimulation to represent the mucus layer. A pro-inflammatory environment was created by combining the co-culture with LPS-activated dendritic cells (DCs) in the basolateral compartment. The presence of activated DCs caused a decrease in transepithelial electrical resistance (TEER), which was slightly restored by LCA and sulfated DCA. The expression of genes related to intestinal epithelial integrity and the mucus layer were slightly, but not significantly increased. These results imply that sulfated BAs have a minor effect on intestinal barrier function in Caco-2 and HT29-MTX-E12 cells. When exposed directly to DCs, our results point towards anti-inflammatory effects of secondary BAs, but to a minor extent for sulfated secondary BAs. Future research should focus on the importance of proper transformation of BAs by bacterial enzymes and the potential involvement of BA dysmetabolism in IBD progression.

## 1 Introduction

Inflammatory bowel disease (IBD) comprises a set of disorders that causes chronic and relapsing inflammation of the gastrointestinal tract. The etiology of IBD remains largely unknown, although it is clear that it is a multifactorial disease, in which the complex interplay between genetic susceptibility, environmental stimuli and the immune system are involved [1, 2]. Furthermore, the gut microbiota is thought to play a major role in the onset and progression of IBD, which is emphasized by studies showing that gut microbiota composition in IBD patients is dysbiotic [3, 4, 5, 6]. Dysbiosis is linked to disturbed intestinal barrier function, such as increased intestinal permeability [7] and an impaired mucus layer [8, 9]. Impaired intestinal barrier function enables direct bacterial contact with the epithelial cell layer, thereby inducing an inflammatory response [10, 11, 12, 13]. In a healthy situation, the intestinal mucosal immune system is tolerant against commensal bacteria, a process in which intestinal dendritic cells (DCs) play a crucial role [14, 15, 16]. During IBD, intestinal DCs have lost their tolerogenic function and produce elevated levels of pro-inflammatory cytokines, consequently leading to an exacerbated disease progression [17, 18].

Importantly, dysbiosis is also linked to an altered production of bacterial metabolites, such as secondary bile acids (BAs) [3, 19, 20, 21]. Primary BAs are synthesized in the liver, conjugated with taurine or glycine and secreted in the small intestine, where they accomplish a major role in lipid digestion [22]. BAs are actively reabsorbed in the ileum, transported back to the liver and metabolized by hepatic enzymes to be reused again, which is a process called the enterohepatic cycle [22]. Approximately 5% of all BAs are not reabsorbed and enter the colon, where resident bacteria deconjugate and metabolize them into secondary BAs. These secondary BAs can be either excreted via feces or reabsorbed and transported back to the liver. However, secondary BAs might be hepatotoxic at high concentrations and are therefore first detoxified by addition of a sulfonate group (SO^3-^) [23]. As a result of IBD-related dysbiosis, the production of bacterial enzymes and thus BA metabolism can be disturbed, a process known as BA dysmetabolism [24]. Indeed, the capacity of the gut microbiota to deconjugate BAs and transform primary to secondary BAs was decreased in patients with active IBD. As a consequence, increased abundance of conjugated BAs and decreased abundance of secondary BAs in feces of IBD patients during both remission and active disease was detected, as compared to healthy people [3, 25]. Similar differences in BA composition were found in other studies investigating fecal metabolite pools in IBD patients [19, 20, 26, 27]. Interestingly, dysbiosis in IBD patients was also associated with a reduced desulfation capacity, which was concomitant with 15% higher levels of fecal sulfated BAs [3]. Likewise, increased levels of fecal 3-sulfodeoxycholic acid and chenodeoxycholic acid sulfate were found in Crohn's disease patients [20]. The fecal abundance of sulfated BAs was also found to be elevated in patients with non-inflammatory intestinal disorders, such as diarrhea-predominated irritable bowel syndrome [25, 28].

Given the important signaling functions of secondary BAs, including their role in inflammatory pathways, a change in luminal BA composition may have consequences on the progression of IBD. However, the possible involvement of sulfated BAs is only based on associative studies and the causal effects remain elusive. Therefore, the aim of this study was to investigate the effects of sulfated BAs on intestinal barrier function and immune response. Since existing models often insufficiently approach the physiological representation of the intestinal barrier and inflammatory environment in the context of IBD, we first established a novel inflammatory *in vitro* human intestinal model. We included a co-culture of Caco-2 and HT29-MTX-E12 cells, which are both human colon carcinoma cell lines representing an enterotype and a mucus-producing cell line, respectively. To mimic the inflammatory state as observed during IBD, the co-culture was grown on cell culture inserts in combination with DCs in the basolateral compartment, which were activated with LPS to obtain pro-inflammatory properties. In contrast to existing models, our model had an improved mucus layer by growing the cells on a semi-wet interface with mechanical stimulation (SMWS) [29, 30]. After exposure to sulfated deoxycholic acid (DCA), sulfated lithocholic acid (LCA) and their unsulfated forms for 24 h, the effects on intestinal barrier function and immune response were investigated. New insights into the role of BA dysmetabolism in IBD may contribute to the discovery of novel therapies that may add to the treatment of IBD.

## 2 Materials and methods

### 2.1 Cell culture

Caco-2 cells (ATCC) and HT29-MTX-E12 cells (ECACC) were cultured in Dulbecco's Modified Eagle Medium supplemented with 10% Fetal Bovine Serum and 1% penicillin/streptomycin. Cells were grown until 80–90% confluence at 37 ​°C/5% CO_2_. Passage numbers between 7 and 25 were used for Caco-2 cells and between 3 and 15 for HT29-MTX-E12 cells. Monocytes were isolated from buffy coats originated from different blood donors (Sanquin, Nijmegen, The Netherlands). First, PBMCs were isolated from the buffy coat using LeucoSep tubes (Greiner-Bio One, Alphen aan den Rijn, The Netherlands), pre-filled with Ficoll-Paque Plus (GE Healthcare via Sigma-Aldrich). PBMCs were filtered through a 70 μm cell strainer (Corning) and counted using a Vi-Cell counter (Beckman Coulter, Woerden, The Netherlands). A QuadroMACS Separator (Miltenyi Biotec, Leiden, The Netherlands) was used to magnetically separate CD14^+^ monocytes, using MojoSort Human CD14 Nanobeads (BioLegend, London, UK) diluted in MACS buffer (PBS, 0.5% BSA and 2mM EDTA) following the manufacturer's instructions. Monocytes were resuspended in RPMI, supplemented with 10% FCS, 1% penicillin/streptomycin and 1% GlutaMAX (Gibco). Monocytes were differentiated into dendritic cells by adding 10 ng/mL Granulocyte macrophage-colony stimulating factor (GM-CSF) (Miltenyi Biotec, Leiden, The Netherlands) and 10 ng/mL human recombinant IL-4 (PeproTech, London, UK) for 6 days at 37 ​°C/5% CO_2_.

### 2.2 Cell model

A co-culture of Caco-2 cells and HT29-MTX-E12 cells was seeded in 24-well ThinCert cell culture inserts with 0.4 μm pores (Greiner-Bio One, Alphen aan den Rijn, The Netherlands). Caco-2 and HT29-MTX-E12 cells were seeded in a 3:1 ratio, using a seeding density of 225,000 cells/mL in a volume of 150 μL. A volume of 700 μL DMEM was added to the basolateral compartment. Two days after seeding, media volumes were changed to 25 μL and 425 μL in the apical and basolateral compartment, respectively. The cell culture plates were put on a CO_2_ resistant shaker (Thermo Fisher Scientific, Breda, The Netherlands) at 65 rpm. Cells were differentiated for 14 days and medium was changed every other day. Immature DCs were seeded in 24-wells plates in a density of 400,000 cells per well. DCs were stimulated with 10 ng/mL LPS (L3024, Sigma-Aldrich, Darmstadt, Germany) for 24 h. Maturation of DCs was checked on the CytoFLEX Flow Cytometer (Beckman Coulter, Woerden, The Netherlands) using CD14-ECD antibody, clone RMO52 (IM2707 ​ ​ ​U, Beckman Coulter), FITC anti-human CD83, clone HB15e and PE/Cyanine7 anti-human CD209 (DC-SIGN), clone 9E9A8 antibodies (BioLegend, Amsterdam, The Netherlands). The culture inserts with Caco-2 and HT29-MTX-E12 cells were transferred to the cell culture plate containing the LPS-activated DCs, without changing of the LPS-containing DC culture medium. The co-culture was exposed to lithocholic acid 3-sulfate disodium salt (sulfo-LCA) (Santa-Cruz Biotechnology, Dallas, United States), deoxycholic acid 3-O-sulfate disodium salt (sulfo-DCA) (Toronto Research Chemicals, Toronto, Canada), lithocholic acid (LCA) and deoxycholic acid (DCA) (Sigma-Aldrich, Darmstadt, Germany). LCA and sulfo-LCA were solubilized in DMEM:methanol (1:1, v/v). DCA and sulfo-DCA were solubilized in DMEM:methanol (3:1, v/v). The concentrations of BAs used were based on physiological concentrations [3]. A control without DCs and a control with LPS-activated DCs were included. Control cells were exposed to similar concentrations of methanol (0.5%). Every condition was applied in duplicate. A total of three similar plates were seeded and exposed to BAs; plate 1 was used for permeability assays, plate 2 for RNA isolation and plate 3 three for protein isolation. Experiments where DCs were directly exposed to BAs were performed similarly, except that BAs were applied directly to the DCs.

### 2.3 Quantification of lactate hydrogenase release

To investigate the effects of BA exposure on cytotoxicity of Caco-2 and HT29-MTX-E12 cells and DCs, lactate hydrogenase levels were measured in conditioned medium collected directly after 24 h of BA exposure. To this end, a lactate dehydrogenase (LDH) cytotoxicity detection kit (Roche Applied Science; Almere, The Netherlands) was used following the manufacturer's instructions. As a control for complete cytotoxicity, cells were exposed for 15 min to a 1% Triton-X100 solution.

### 2.4 Trans- and paracellular epithelial permeability assays

Transepithelial resistance (TEER) was measured with an EVOM2 Volt/Ohm meter using STX2 electrodes (World Precision Instruments, Sarasota, United States). To assure the electrodes were fully submerged in medium, the media volumes were adapted to 100 μL apical and 700 μL basolateral before the first TEER measurements were performed. The TEER values after BA exposure were expressed as percentage of the TEER value measured just before BA exposure. After 24 h of BA exposure, culture inserts were washed twice with PBS and transferred to a new 24-wells plate. Lucifer Yellow CH dilithium salt (L0259, Sigma) was dissolved in phenol red-free medium (Gibco) to 1 mg/mL and 100 μL was added to the apical compartment. In the basolateral compartment, 700 μL phenol red-free DMEM was added and afterwards the plate was incubated at 37 ​°C/5% CO_2_ for 3 h. Subsequently, 100 μL of the basolateral compartment was collected and fluorescence was measured at 425/515 nm (excitation/emission). An empty cell culture insert served as a control for complete paracellular permeability.

### 2.5 RNA isolation and qRT-PCR

The cell culture inserts of plate 2 were washed twice with ice-cold PBS and subsequently, 200 μL TRIzol reagent (ThermoFisher) was added per insert. The duplicates per condition were pooled to assure enough RNA yield. RNA was isolated using phenol/chloroform extraction. The RNA concentration was measured using a Nanodrop (Nanodrop ND-1000, Nanodrop Products, Maarssen, The Netherlands). A total of 1000 ng RNA was reverse transcribed using the RevertAid First Strand cDNA Synthesis kit (ThermoFisher). Real-time quantitative PCR was carried out using the SensiMix SYBR kit (Bioline, Alphen aan den Rijn, The Netherlands) in a CFX384 machine (Bio-Rad). Primer sequences are listed in Table 1. Data was normalized against the housekeeping gene *GAPDH*.

**Table 1:** Primer sequences used for qRT-PCR.

Gene	Forward primer	Reverse primer
*GAPDH*	GAAGGTGAAGGTCGGAGTC	GAAGATGGTGATGGGATTTC
*OCLN*	CGGCGAGTCCTGTGATGAG	TCTTGTATTCCTGTAGGCCAGT
*ZO1*	GAACGAGGCATCATCCCTAA	CCAGCTTCTCGAAGAACCAC
*CDH1*	CGACCCAACCCAAGAATCTA	AGGCTGTGCCTTCCTACAGA
*CLDN1*	CTTTGGGGCTTTGATCGGACT	GGAGTAGTTCAATTCCAGCAACA
*MUC2*	ACCCGCACTATGTCACCTTC	GGACAGGACACCTTGTCGTT
*MUC5AC*	CAGCACAACCCCTGTTTCAAA	GCGCACAGAGGATGACAGT
*DEFB1*	ATGAGAACTTCCTACCTTCTGCT	TCTGTAACAGGTGCCTTGAATTT
*LYZ*	GGCCAAATGGGAGAGTGGTTA	CCAGTAGCGGCTATTGATCTGAA
*CA12*	AGTGACATCCTCCAGTATGACG	GTGGCACTGTAGCGAGACT
*ANG*	CCTCCATGCCAGTACCGAG	GGACGACGGAAAATTGACTGA
*ASBT*	TGTGTTGGCTTCCTCTGTCAG	GGCAGCATCCTATAATGAGCAC
*FABP6*	GCCCGCAACTTCAAGATCG	CCTTGCCAACAGTGAACTTGT
*FGF19*	CACCAGGCTTCAGGAGTAGG	CGGGACAGCAAGTTATTCTC
*OSTα*	TCATTTCCCGTCAAGCCAGG	GGCGAACAAGCAATCTGCC
*OSTβ*	TCCAGGCAAGCAGAAAAGAAA	ACTGACAGCACATCTCTCTCT
*SULT2A1*	CTGGGAAAGACGTTAGAACCC	AAGTTGTGCTTTGTCCACTACAT

### 2.6 Protein isolation and western immunoblotting

The cell culture inserts were washed twice with ice-cold PBS and 100 μL RIPA buffer (ThermoFisher) enriched with protease- and phosphatase inhibitors (Roche Diagnostics) was added per culture insert. Duplicates were pooled to assure enough protein yield. Cell lysates were incubated on ice for 20 min following centrifugation for 10 min at 13,000 *g*. Protein concentrations of the supernatants were measured using a bicinchoninic acid assay (ThermoFisher). For each sample, 14.8 μg protein was loaded on a 4–15% Mini-PROTEAN TGX Precast gel (Bio-Rad). Proteins were separated by SDS gel electrophoresis and transferred onto a polyvinylidene difluoride (Trans-Blot Turbo Midi 0.2 μm PVDF Transfer Packs, Bio-Rad) membrane using the Transblot Turbo System (Bio-Rad). After blocking for 1 h at room temperature, the membranes were incubated overnight at 4 °C with anti-ZO1 (Abcam ab216880), anti-OCLN (Abcam ab216327) and anti-HSP90 (Cell Signaling Technology 4874). ZO1 and OCLN antibodies were used in 1:1000 and for HSP90 1:5000 was used. Subsequently, membranes were incubated with HRP conjugated goat anti-rabbit IgG antibody (1:5000) (GenScript A00098) for 1 h at room temperature. All membrane incubations were in Tris-buffered saline with 0.1% Tween 20 (TBS-T) and 5% (w/v) skimmed dry milk. Washing in between steps was done in TBS-T. Blots were visualized with Clarity ECL substrate (Bio-Rad) using the ChemiDoc MP system (Bio-Rad). Quantification was performed using ImageLab software (Bio-Rad).

### 2.7 Cytokine measurements

Medium collected from the basolateral compartments was used for the assessment of cytokines. Levels of IL-6, IL-12/IL-23 p40 and TNF-α were measured with human DuoSet ELISA Development kits (R&D Systems, Abingdon, UK) following the manufacturer's instructions.

### 2.8 Statistical analysis

Data is presented as mean ± standard deviation (SD). GraphPad Prism version 5 (San Diego, CA, USA) was used for the statistical analyses. Differences between the control and BA-exposed groups were determined with an unpaired Student's t-test, unless stated otherwise. A value of *p* ≤ 0.05 was considered as statistically significant. A total of three biological replicates were performed.

## 3 Results

### 3.1 Establishment of an inflammatory *in vitro* human intestinal model consisting of Caco-2 and HT29-MTX-E12 cells combined with LPS-activated dendritic cells

**Figure 1:**
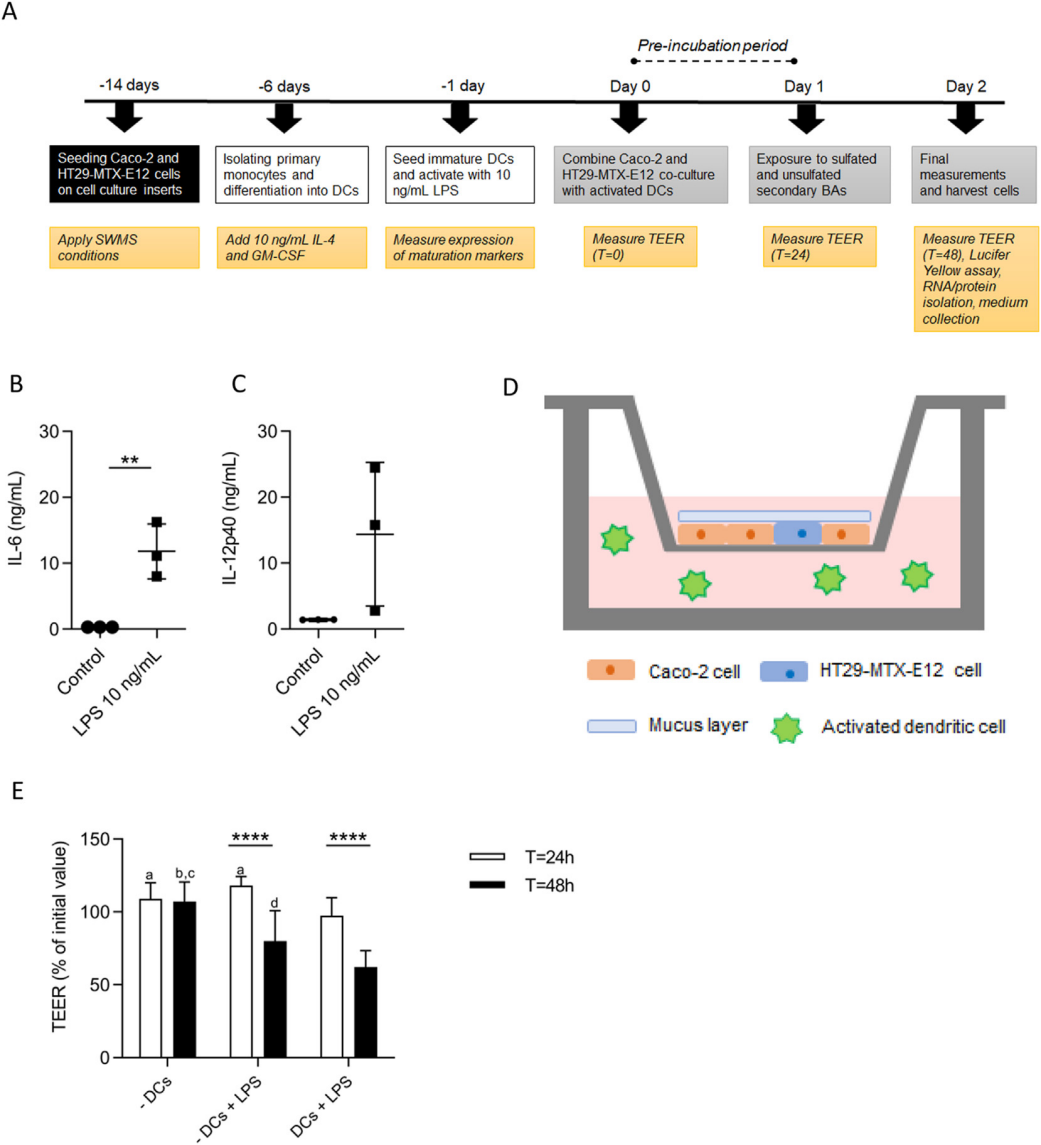
Establishment of a triple co-culture of Caco-2, HT29-MTX-E12 cells combined with activated dendritic cells. (A) Schematic overview of study design in chronological order. (B) Concentrations of IL-6 and (C) IL-12p40 in DC supernatant after activation with 10 ng/mL LPS for 24 h ∗∗*p* < 0.01. (D) Schematic overview of the Caco-2 and HT29-MTX-E12 co-culture grown in a 3:1 ratio on cell culture inserts with a mucus layer on top. Activated DCs are in the basolateral compartment. (E) TEER measurements of Caco-2 and HT29-MTX-E12 culture at 24 and 48 h. TEER values are expressed as percentage of the initial value. First bar pair: control cells without DCs, second bar pair: control cells with 10 ng/mL LPS in the basolateral compartment, third bar pair: cells combined with activated DCs. Statistical differences were determined using a one-way analysis of variance (ANOVA) followed by a Bonferroni *post hoc* test. ^a^*p* < 0.001 at T = 24 ​h compared to condition with activated DCs. ^b^*p* < 0.001 at T = 48 ​h compared to condition without DCs, but with basolateral LPS. ^c^*p* < 0.0001 at T = 48 ​h compared to condition with activated DCs. ^d^*p* < 0.05 at T = 48 ​h compared to condition with activated DCs. ∗∗∗∗*p* < 0.0001. Data are derived from 3 independent biological replicates.

The first important step of this study was to establish an *in vitro* human intestinal model with an improved physiological representation of the intestinal barrier and inflammatory environment in the context of IBD. In Figure 1A, a schematic overview of the study design is given. Caco-2 and HT29-MTX-E12 cells were seeded in a 3:1 ratio on cell culture inserts and SWMS conditions were applied. In parallel, primary monocytes were isolated from three human buffy coats and differentiated into DCs. Activation with 10 ng/mL LPS for 24 h resulted in mature DCs expressing the DC surface markers CD83 and CD209 (Supplementary file 1). Activated DCs produced higher levels of IL-6 (*p* = 0.0088) and IL-12p40 (*p* = 0.1) compared to DCs that were not activated (Figure 1B, C), although IL-12p40 levels of one biological replicate were relatively low (Figure 1C). After 24 h of LPS exposure, the cell culture inserts with the Caco-2/HT29-MTX-E12 co-culture were positioned in the cell culture plates containing activated DCs. This resulted in a model consisting of intestinal cells in the apical compartment and LPS-activated DCs in the basolateral compartment (Figure 1D). TEER values measured at 24 and 48 h after combination with activated DCs decreased with 12 and 45 percentage points, respectively, compared to the condition without basolateral DCs (*p* < 0.001 and *p* < 0.0001) (Figure 1E). In the next BA-exposure experiments, we used a pre-incubation period of 24 h. Altogether, we confirmed that the presence of activated DCs in the basolateral compartment caused a pro-inflammatory state, reflected by the elevated cytokine levels. This likely resulted in the observed increased intestinal permeability of the intestinal cells.

### 3.2 Intestinal permeability was slightly restored by LCA and sulfated DCA under inflammatory conditions

**Figure 2:**
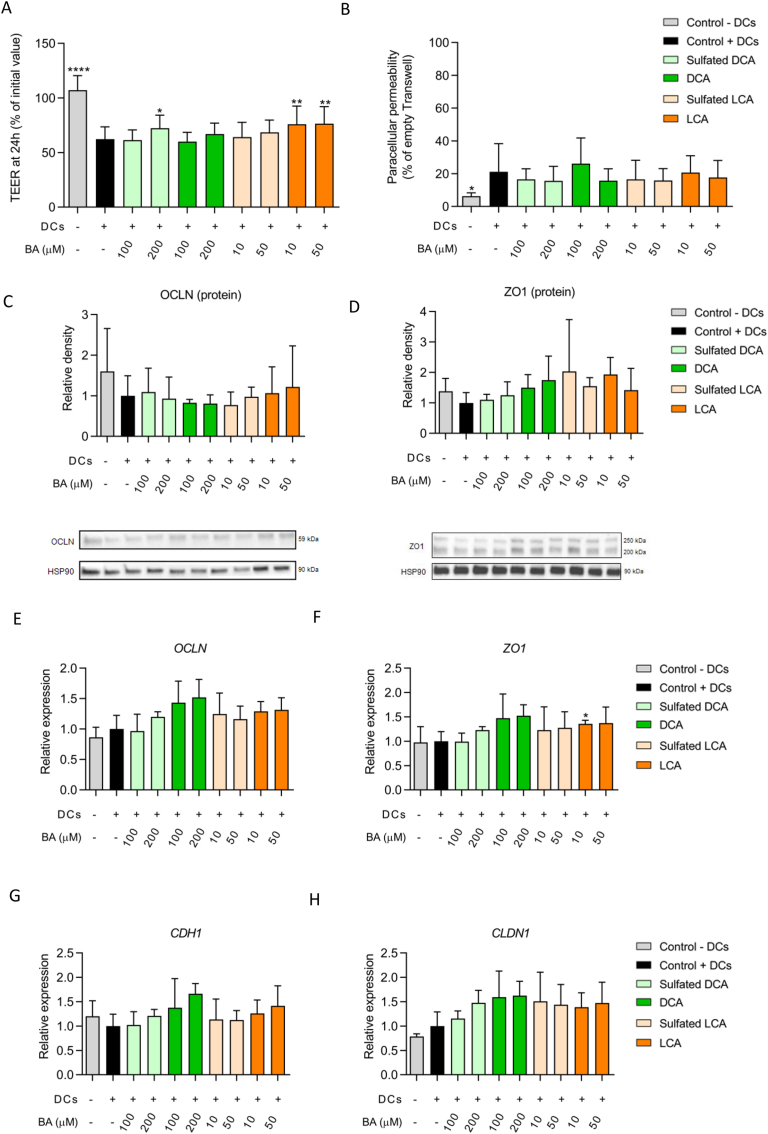
Intestinal permeability assays of Caco-2 and HT29-MTX-E12 cells combined with activated DCs after treatment with (sulfated) BAs for 24h. (A) TEER values expressed as % of initial values. (B) Fluorescence measured in basolateral compartment after apical Lucifer Yellow incubation for 3 h. Values are expressed as % of an empty cell culture insert (representing complete translocation). (C–D) Protein quantity of Occludin (OCLN, 59 kDa) and Zona Occludens-1 (ZO1, 200 kDa) relative to the control. For ZO1, an unspecific band was observed at 250 kDa. The band at 200 kDa was used for quantification. See Supplementary file 2 for uncropped images. (E–H) Panel of genes related to intestinal permeability (*OCLN*: Occludin, *ZO1*: Zonula Occludens-1, *CDH1*: E-cadherin, *CLDN1*: Claudin-1). Expression of proteins and genes of interest is expressed relative to the control (Caco-2 and HT29-MTX-E12 cells exposed to activated DCs in basolateral compartment). ∗p < 0.05; ∗∗p < 0.01; ∗∗∗∗p < 0.0001 compared to the control condition with activated DCs.

After the pre-incubation period, the co-cultures of Caco-2 and HT29-MTX-E12 cells were exposed to sulfated DCA, sulfated LCA and their unsulfated forms in different concentrations for another 24 h. Cytotoxicity measured by the release of LDH in the apical medium was not different between cells exposed to BAs compared to unexposed cells (data not shown). The TEER of all conditions exposed to BAs in the presence of activated DCs were significantly lower compared to the control without DCs (*p* < 0.0001) (Figure 2A). Exposure to sulfated DCA (200 μM) and both concentrations of LCA (10 μM and 50 μM) resulted in a slight, but significant restoration of the TEER (Figure 2A). The same cell culture inserts were subjected to a Lucifer Yellow assay to investigate if BA treatment had an effect on paracellular permeability. The flux of Lucifer Yellow from the apical to basolateral compartment was significantly lower in cells cultured without DCs compared to the control with DCs (*p* < 0.05) (Figure 2B). None of the BAs had a significant additional effect on paracellular permeability.

### 3.3 Expression of genes related to intestinal epithelial integrity tended to increase after BA exposure

To further investigate the effects of sulfated secondary BAs on intestinal barrier function, we measured the expression of proteins related to intestinal epithelial integrity. In line with the significant TEER reduction (Figure 2A), lower protein levels of Occludin (OCLN) and Zonula Occludens-1 (ZO1) were measured in cells exposed to activated DCs compared to the control cells without DCs (Figure 2C,D), but these differences were not significant. Next, we investigated whether these lower protein levels were the result of decreased mRNA levels. However, *OCLN* and *ZO1* mRNA levels were not significantly affected by the presence of activated DCs in the basolateral compartment (Figure 2E,F). Other genes related to intestinal barrier function, E-cadherin (*CDH1*) and Claudin-1 (*CLDN1*), were also not affected (Figure 2G,H). Interestingly, protein levels of OCLN and ZO1 were not affected by BA exposure, whereas expression of *OCLN*, *ZO1*, *CDH1* and *CLDN1* followed an increasing trend after exposure to most BAs, although these differences were not significant (Figure 2C-H). Together, these results indicate that differences in intestinal barrier function measured by TEER were partly reflected at gene and protein level.

### 3.4 Differential expression of FXR-target genes by unsulfated, but not sulfated secondary BAs

**Figure 3:**
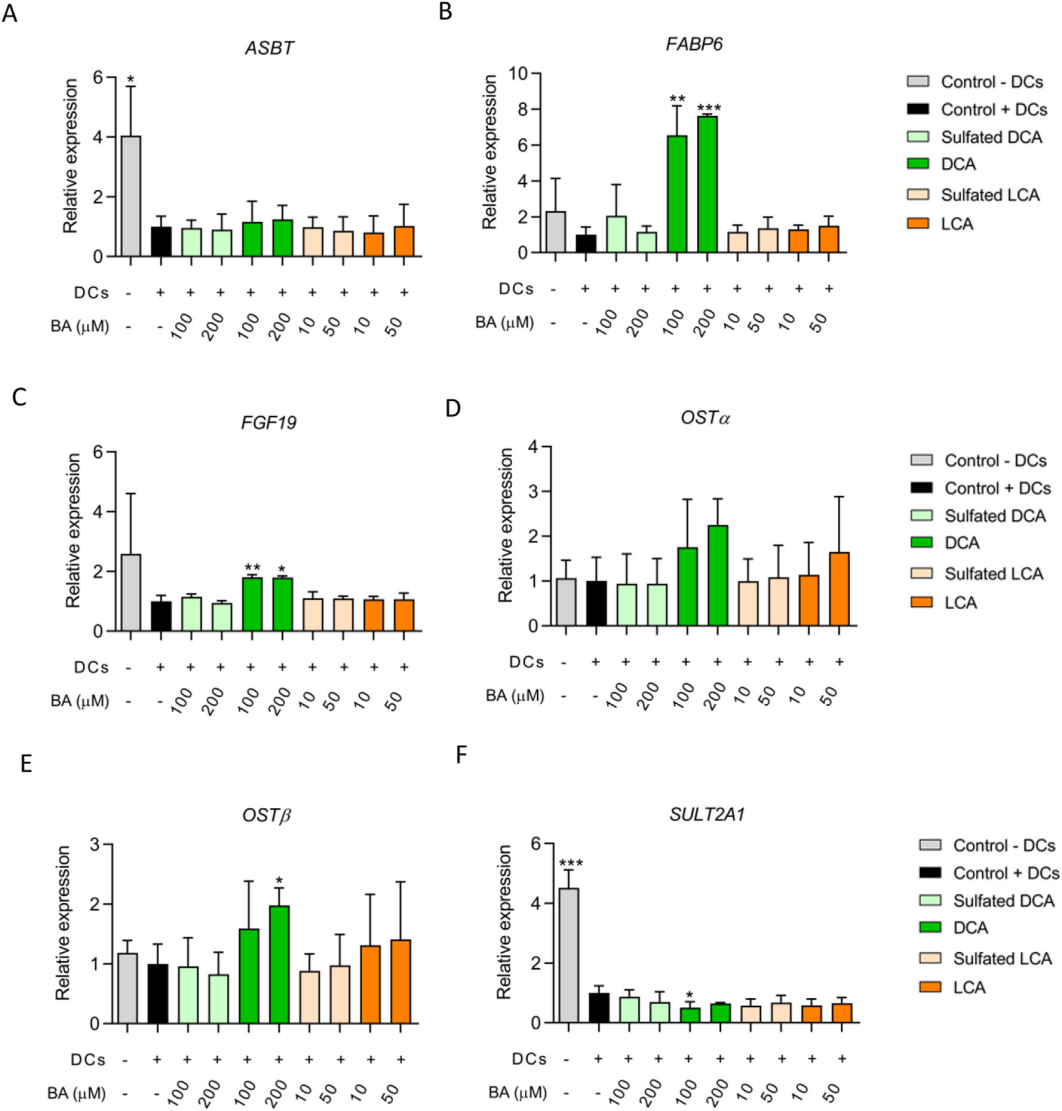
Expression of FXR target genes. (A) Apical bile salt transporter (*ASBT*), (B) Ileal bile acid binding protein (*FABP6*), (C) Fibroblast growth factor 19 (*FGF19*), (D) Basolateral organic solute transporter alpha, (E) basolateral organic solute transporter beta (*OSTα/β*), and (F) Sulfotransferase family 2A member 1 (*SULT2A1*). Expression of genes of interest is expressed relative to the control (Caco-2 and HT29-MTX-E12 cells exposed to activated DCs in basolateral compartment). ∗*p* < 0.05, ∗∗*p* < 0.01, ∗∗∗*p* < 0.001 compared to condition with activated DCs.

Next, we aimed to find out if exposure to sulfated and unsulfated secondary BAs resulted in activation of FXR. While DCA and LCA are potent activators of FXR [22], it is unknown whether the sulfated forms of these BAs also activate FXR, as these BAs are not, or poorly absorbed by enterocytes [23]. To this end, we investigated if exposure to DCA, LCA and their sulfated forms resulted in differential expression of a selection of FXR-target genes: ileal bile acid binding protein (*IBABP, FABP6*), fibroblast growth factor 19 (*FGF19*), basolateral organic solute transporters alpha and beta (*OSTα/β, SLC5*1A/B), apical bile salt transporter (*ASBT, SLC10A2*) and sulfotransferase family 2A member 1 (*SULT2A1*) [31, 32, 33, 34]. Interestingly, the addition of activated DCs potently reduced the expression of *ASBT* (*p* < 0.05) and *SULT2A1* (*p* < 0.001) (Figure 3A, F). *ASBT* was not differentially expressed by any of the BAs (Figure 3A). In contrast, *FABP6*, *FGF19* and *OSTβ* were significantly upregulated in cells exposed to DCA compared to the control with activated DCs (Figure 3B,C, E). Interestingly, exposure to 100 μM DCA reduced *SULT2A1* expression compared to the control cells with DCs (Figure 3F). Altogether, these results indicate that DCA had pronounced effects on the expression of most FXR-target genes, while LCA did not have a significant effect. Exposure to neither sulfated DCA nor sulfated LCA resulted in a differential expression of any FXR-target genes. Importantly, mRNA levels of *ASBT* and *SULT2A1* were significantly decreased by the presence of basolateral activated DCs.

### 3.5 No effects of sulfated secondary BAs on *MUC2* and *MUC5AC* expression

**Figure 4:**
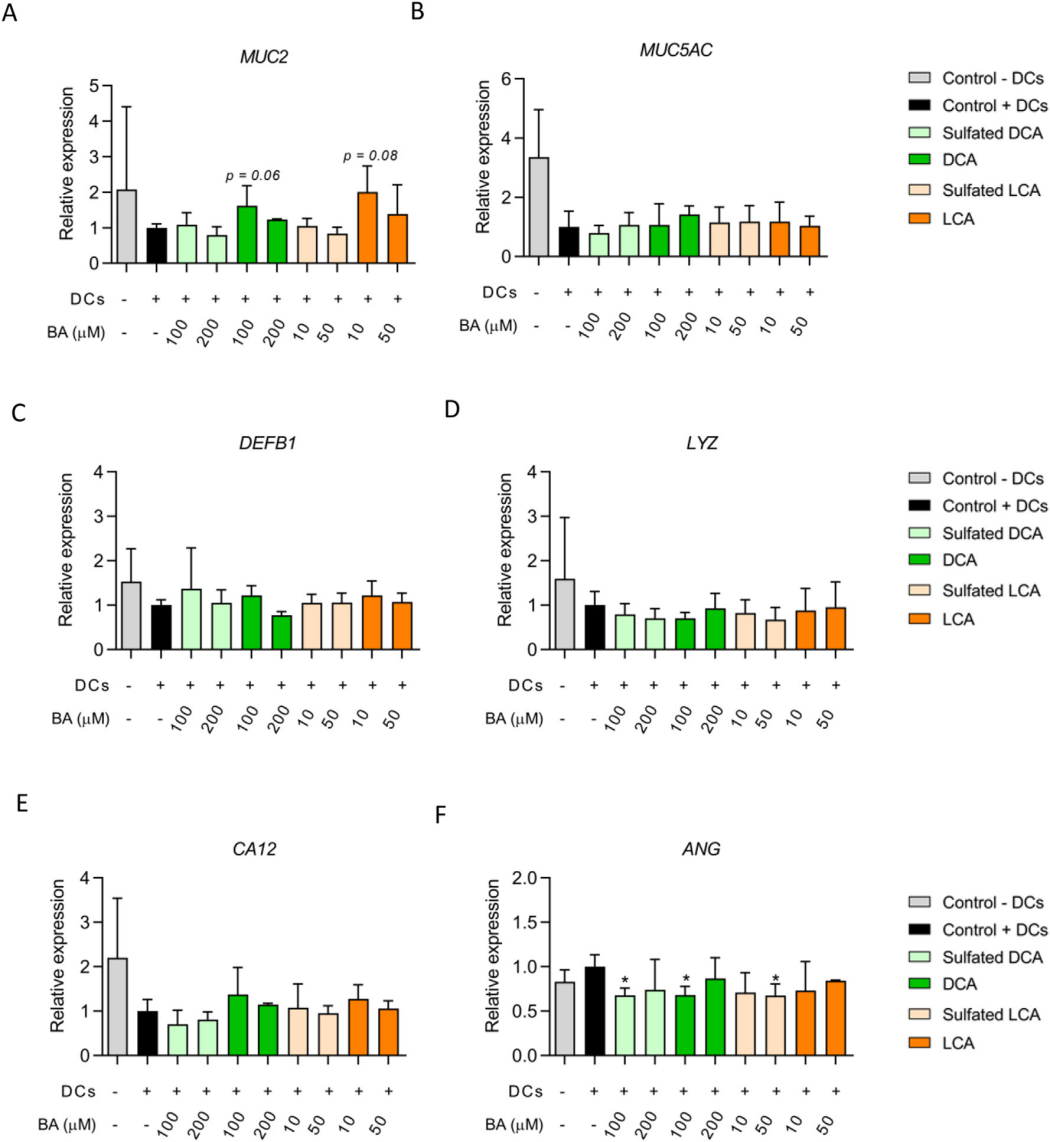
Expression of mucin and antimicrobial peptides. A) Expression of Mucin 2 (*MUC2*) and B) Mucin 5AC (*MUC5AC*) and genes encoding the antimicrobial peptides (C–F) defensin β-1 (*DEFB1*), lysozyme (*LYZ*), carbonic anhydrase 12 (*CA12*) and angiogenin (*ANG*). Expression of genes of interest is expressed relative to the control (Caco-2 and HT29-MTX-E12 cells exposed to activated DCs in basolateral compartment). ∗*p* < 0.05 compared to condition with activated DCs.

In order to determine if sulfated secondary BAs had an effect on the mucus layer, we investigated the expression of *MUC2*, which is the most dominant gel-forming mucin present in the intestine. Moreover, we also measured expression of *MUC5AC.* This is another gel-forming mucin which is normally not secreted in the intestine, but is secreted in HT29-MTX-E12 cells, even after growing this cell type under SWMS conditions [29, 30]. Interestingly, the presence of activated DCs decreased the expression of *MUC2* and *MUC5AC*, although this effect was not statistically significant (Figure 4A,B). Compared to the control with activated DCs, the expression of *MUC2* seemed to increase after exposure to 100 μM DCA and 10 μM LCA, which was borderline significant (*p* = 0.06 and *p* = 0.08), respectively (Figure 4A). Sulfated BA exposure did not have any effect on mucin mRNA expression.

### 3.6 Subtle effect of some BAs on expression of genes encoding for antimicrobial peptides

Antimicrobial peptides (AMPs) play an important role in intestinal innate immune defense and are known to be produced by enterocytes [35]. We measured the expression of genes encoding the AMPs defensin β-1 (*DEFB1*) and lysozyme (*LYZ*), but also angiogenin (*ANG*) and carbonic anhydrase 12 (*CA12*), since the latter two AMPs are regulated by the BA receptor FXR [36, 37]. Exposure to BAs caused slight, but non-significant changes compared to the control with activated DCs (Figure 4C-F). Only *ANG* was significantly lower expressed after exposure to both 100 μM sulfated DCA and DCA, as well as 50 μM LCA (*p* < 0.05) (Figure 4F).

### 3.7 No indirect effects of BA exposure on cytokine production by basolateral DCs

**Figure 5:**
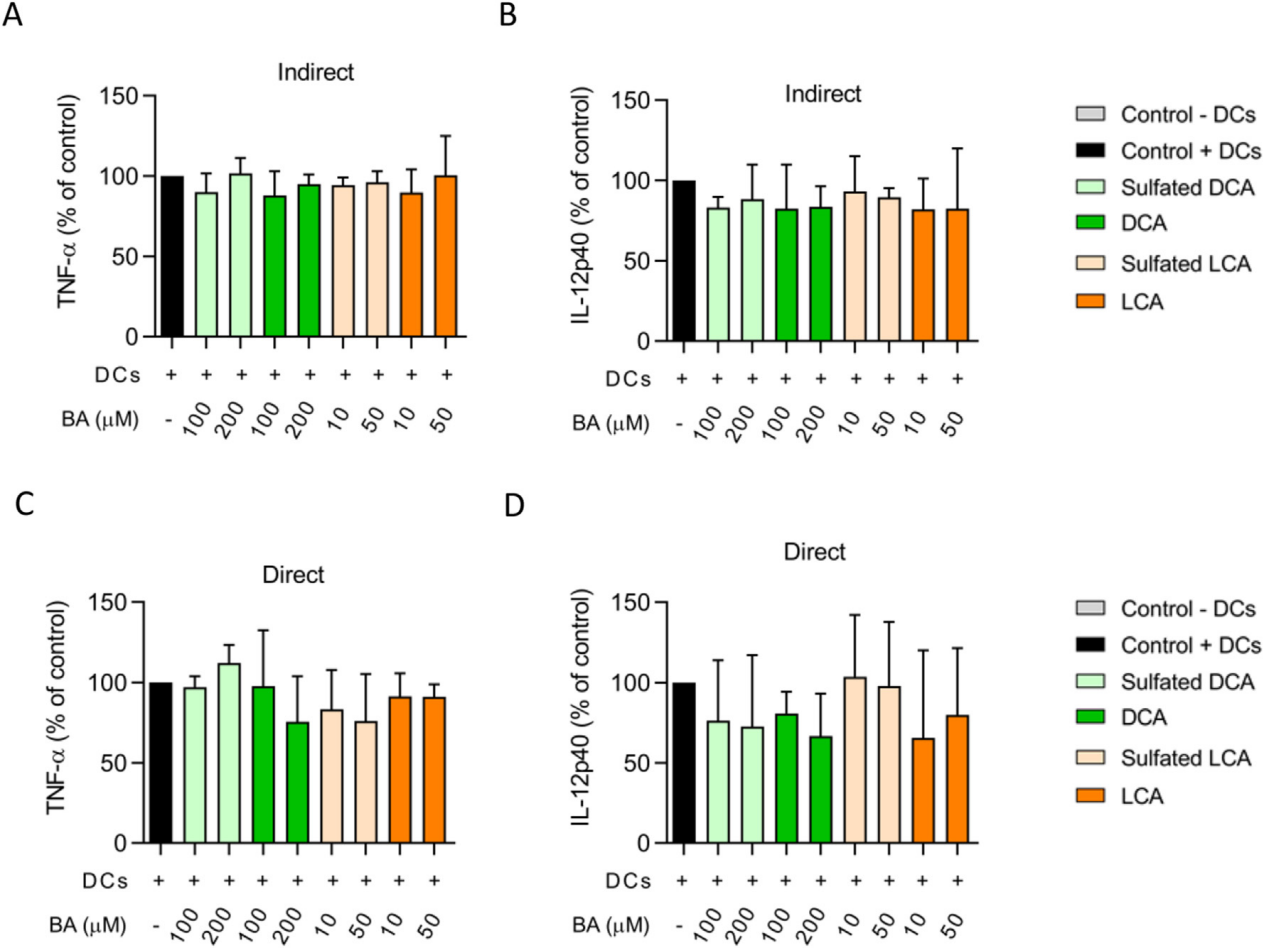
Cytokine levels produced by basolateral DCs after indirect BA exposure (via cell culture inserts) and direct exposure. A) TNF-α and B) IL-12p40 levels after indirect exposure. C) TNF-α and D) IL-12p40 levels after direct exposure. Cytokine levels are expressed as percentage of the control, i.e. DCs exposed to only medium.

Although the presence of activated DCs in the basolateral compartment resulted in a significant increase in permeability of the Caco-2/HT29-MTX-E12 co-culture, apical exposure to sulfated and unsulfated secondary BAs did not have a major additional effect on intestinal epithelial integrity (Figure 2A,B). We hypothesized that BAs might have migrated from the apical to the basolateral compartment via the openings between the intestinal epithelial cells, caused by the increased intestinal permeability. In that case, BAs might have come in contact with the DCs present in the basolateral compartment. Therefore, we investigated if this potential indirect contact between BAs and DCs caused an altered immune response by DCs. To this end, TNF-α and IL-12p40 levels were measured in conditioned medium from basolateral DCs after apical exposure to the different BAs. No differences in either TNF-α or IL-12p40 levels were found (Figure 5A,B).

### 3.8 Decreasing, but no significant trend in TNF-α and IL-12p40 production by activated DCs after direct exposure to secondary BAs

The finding that cytokine production by DCs was not affected by indirect BA exposure could either indicate that BAs were not migrated towards the basolateral compartment, or that DCs were not affected by BA exposure in terms of TNF-α and IL-12p40 production. To investigate if direct exposure to BAs caused an effect on immune response in DCs, we exposed activated DCs directly to sulfated and unsulfated secondary BAs under similar conditions as previous experiments with indirect exposure. DCA caused a decrease in both TNF-α and IL-12p40 levels compared to the control cells (Figure 5C,D), but these differences were not significant. Lower IL-12p40 levels were found after LCA exposure, albeit variation between biological replicates was high (Figure 5D, Supplementary file 1). No significant differences were found after exposure to sulfated BAs.

## 4 Discussion

The rising prevalence of IBD in many countries is alarming, given the concomitant increase in social and economic burden associated with this disease [38]. To decrease this burden, it is of utmost importance to better understand the underlying causes of IBD, especially because the etiology of IBD is still largely unknown. Emerging evidence suggests a potential role for BA dysmetabolism in IBD, however, the exact effects of elevated levels of IBD-associated BA subtypes are not widely investigated yet. In the present study, we aimed to investigate the effects of sulfated secondary BAs on intestinal barrier function in the context of IBD. Furthermore, we also investigated if sulfated BAs had an effect on immune response in human monocyte-derived DCs.

We first aimed to establish an inflammatory *in vitro* human intestinal model, as existing models often insufficiently reflect the chronic inflammatory state in the context of IBD. For example, many existing models either add a cytokine cocktail to induce a pro-inflammatory state [39, 40] or use THP-1 cells as representation of immune cells [41, 42, 43, 44, 45, 46]. The effectiveness of this cell line in an intestinal model is questionable. In two studies, Caco-2 cells exposed to THP-1 cells were severely damaged after 48 h, which was reflected by the high cytotoxicity values and TEER decrease of more than 80% [44, 46]. Given the crucial role of intestinal DCs in IBD pathophysiology [14, 47], we used human monocyte-derived DCs in our model. After activation with LPS, these DCs produced high cytokine levels, resulting in an increased epithelial permeability without affecting cytotoxicity. To improve the physiological representativeness of our model even more, we also paid special attention to the mucus layer, since it is often underrepresented or even lacking in most existing intestinal *in vitro* models. Therefore, we cultured the Caco-2/HT29-MTX-E12 co-culture under SWMS conditions, which was shown to improve the quantity and quality of the mucus layer [29, 30]. Importantly, the use of *in vitro* models has some limitations, e.g. with regard to the translatability of the *in vivo* situation. However, we deemed our model suitable at this, more explorative phase, of our study.

After successful optimization, we exposed the inflammatory *in vitro* human intestinal immune model to sulfated and unsulfated secondary BAs for 24 h and investigated the effects on intestinal barrier function. We found a slight TEER restoration after exposure to LCA and sulfated DCA, but not DCA and sulfated LCA. These effects on intestinal epithelial barrier integrity were partly reflected at protein level. Previous *in vitro* studies also showed TEER restoration by LCA in the presence of inflammatory conditions [40, 48]. With regard to DCA, we did not find an effect on TEER, while a marked increased permeability caused by DCA was observed in several *in vitro* models [49, 50, 51, 52] as well as in mice [51, 53, 54]. Importantly, we confirmed successful administration of DCA by measuring differential expression of FXR-target genes. Differences in incubation duration and BA concentrations might have impeded direct comparison to existing literature and results of the current study.

In line with the minor effects on intestinal epithelial barrier integrity, we did not find an effect of sulfated BAs on *MUC2* and *MUC5AC* expression. On the contrary, DCA and LCA exposure resulted in an increased expression of *MUC2*, which was borderline significant. As MUC2 plays a crucial role in intestinal barrier protection [55, 56, 57, 58], increased *MUC2* mRNA expression might indicate that these BAs have a restorative effect on the mucus layer. In several human colon cancer cell lines, DCA also caused increased *MUC2* expression [59, 60], but no effects of LCA on mucin mRNA expression have been described. Importantly, it was previously shown that prolonged exposure to pro-inflammatory cytokines strongly decreased mucin gene expression [61, 62]. These results are in line with the decreasing trend in *MUC2* and *MUC5AC* expression that we found after exposure to activated DCs, although this effect was not significant. Next to the effects of BAs on the mucus barrier, it is also important to consider other intestinal barrier properties, such as AMPs that are excreted in the mucus layer [63]. Although DCA was previously shown to increase the expression and secretion of *DEFB1*/DEFB1 *in vitro* [64], we were not able to reproduce these results. We did find a slightly reduced expression of *ANG* by some BAs, which might imply that these BAs have a negative effect on mucosal defense [65]. However, the effects of BAs on AMPs are underexplored in current literature, indicating that more research is needed in this field.

Secondary BAs could have anti-inflammatory effects during intestinal inflammation [27, 66, 67]. Since intestinal DCs are able to sample luminal content [47, 68], we hypothesized that luminal BAs could come in contact with DCs, which might result in an altered immune response. Indeed, direct exposure to secondary BAs caused a decreasing trend in cytokine production, but this effect was less visible after exposure to sulfated secondary BAs. This finding might suggest that increased levels of sulfated BAs at the expense of secondary BAs could abolish the anti-inflammatory effects of secondary BAs. Similar effects were previously found in Caco-2 exposed to sulfated LCA [3], although this effect was found after exposure to relatively high concentrations of LCA and sulfated LCA (400 and 500 μM), which might hamper the physiological translatability of these results.

Here, we present a novel and physiological relevant *in vitro* human intestinal model representing a pro-inflammatory state, which can be used to study intestinal barrier function in the presence of intestinal inflammation. We used this model to investigate the effects of sulfated and unsulfated secondary BAs on intestinal barrier function and immune response in DCs. We show that these BAs had ambiguous effects on intestinal barrier integrity, as reflected by the minor effects on TEER, expression of intestinal epithelial integrity related genes, AMPs and *MUC2*. Although more research is needed, our results hint towards anti-inflammatory effects of secondary BAs, but not sulfated secondary BAs on activated DCs. Future research should focus on the relevance of proper bacterial desulfation activity to assure the anti-inflammatory effects of secondary BAs.

## Declarations

### Author contribution statement

Benthe van der Lugt: Conceived and designed the experiments; Performed the experiments; Analyzed and interpreted the data; Wrote the paper.

Maartje C.P. Vos, Mechteld Grootte Bromhaar: Performed the experiments.

Noortje Ijssennagger, Jocelijn Meijerink, Wilma T. Steegenga: Conceived and designed the experiments.

Frank Vrieling: Conceived and designed the experiments; Contributed reagents, materials, analysis tools or data.

### Funding statement

This work was supported by the 10.13039/501100001720Nutricia Research Foundation with grant no. 2018-25. Benthe van der Lugt was supported by the 10.13039/501100003246NWO Graduate Programme on Food Structure, Digestion and Health (022.006.009). Noortje Ijssennagger was supported by the 10.13039/501100008359MLDS career development grant (CDG16-04) and by the Wilhelmina Children’s Hospital Research Fund.

### Data availability statement

Data included in article/supplementary material/referenced in article.

### Declaration of interests statement

The authors declare no conflict of interest.

### Additional information

No additional information is available for this paper.
