# Supplementary material for: The effects of sulfated secondary bile acids on intestinal barrier function and immune response in an inflammatory in vitro human intestinal model
Source: Heliyon. 2022 Feb 2;8(2):e08883. doi: 10.1016/j.heliyon.2022.e08883 (PMC8829581; doi:10.1016/j.heliyon.2022.e08883)
Supplement: Supplementary file 1 [file mmc1.docx]

**Supplementary file 1**

**
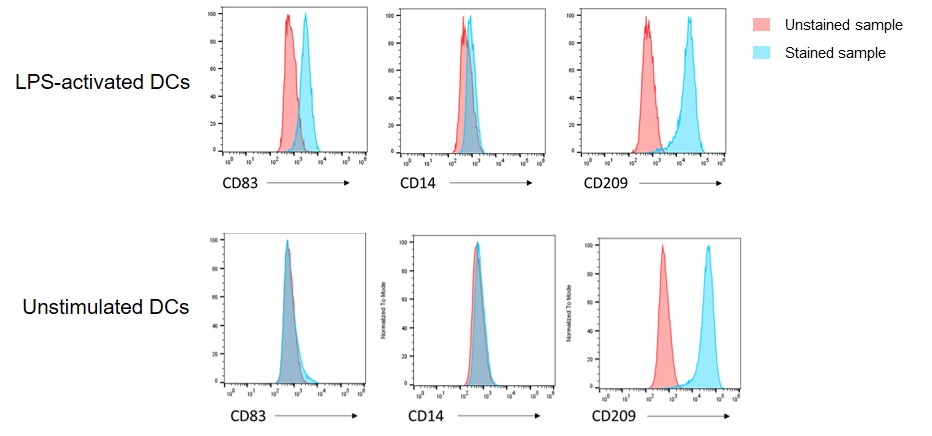
**

**Figure 1** Expression of the cell surface markers CD83, CD14 and CD209 on DCs activated with 10 ng/mL LPS for 24 hours and unstimulated DCs. Unstained (red) and stained (blue) samples are displayed from one representative donor.

**Table 1** **A)** TNF-α levels and **B)** IL-12p40 levels produced by basolateral DCs after direct BA exposure, expressed as percentage of control. Values of all three biological replicates (batch 1, 2, 3) are shown.

**A)**

|  |  | **Sulfo-DCA** | | **DCA** | | **Sulfo-LCA** | | **LCA** | |
| --- | --- | --- | --- | --- | --- | --- | --- | --- | --- |
| **Batch** | **Control** | **100 µM** | **200 µM** | **100 µM** | **200 µM** | **10 µM** | **50 µM** | **10 µM** | **50 µM** |
| 1 | 100 | 93.9 | 124.7 | 134.0 | 93.2 | 87.6 | 87.8 | 92.9 | 88.9 |
| 2 | 100 | 105.0 | 103.4 | 64.9 | 42.7 | 56.8 | 42.8 | 76.4 | 84.5 |
| 3 | 100 | 92.1 | 108.4 | 94.7 | 90.7 | 105.5 | 97.7 | 105.0 | 99.8 |

**B)**

|  |  | **Sulfo-DCA** | | **DCA** | | **Sulfo-LCA** | | **LCA** | |
| --- | --- | --- | --- | --- | --- | --- | --- | --- | --- |
| **Batch** | **Control** | **100 µM** | **200 µM** | **100 µM** | **200 µM** | **10 µM** | **50 µM** | **10 µM** | **50 µM** |
| 1 | 100 | 80.1 | 59.9 | 66.0 | 36.5 | 59.7 | 52.3 | 39.4 | 51.8 |
| 2 | 100 | 36.9 | 35.5 | 92.3 | 78.3 | 121.6 | 121.5 | 28.4 | 60.4 |
| 3 | 100 | 111.9 | 122.2 | 84.1 | 85.3 | 129.9 | 120.3 | 128.4 | 127.7 |
