# Supplementary material for: The effects of sulfated secondary bile acids on intestinal barrier function and immune response in an inflammatory in vitro human intestinal model
Source: Heliyon. 2022 Feb 2;8(2):e08883. doi: 10.1016/j.heliyon.2022.e08883 (PMC8829581; doi:10.1016/j.heliyon.2022.e08883)
Supplement: Supplementary file 2 [file mmc2.pdf]

Supplementary file 2

Occludin (OCLN)

BLOT 1

1. Marker
2. Control – DCs (1.1)*
3. Control + DCs (1.1)*
4. Sulfated DCA 100 uM (1)
5. Sulfated DCA 200 uM (1)
6. Sulfated LCA 10 uM (1)
7. Sulfated LCA 50 uM (1)
8. DCA 100 uM (1)
9. DCA 200 uM (1)
10. LCA 10 uM (1)
11. LCA 50 uM (1)
12. Control + DCs (2.1)*
13. Sulfated DCA 100 uM (2)
14. Sulfated DCA (200 uM) (2)
15. Sulfated LCA 10 uM (2)

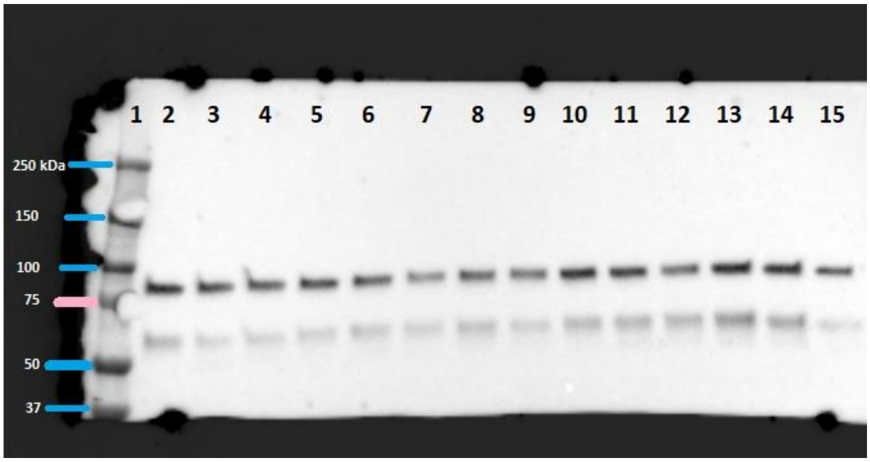

BLOT 2

1. Marker
2. Sulfated LCA 50 uM (2)
3. DCA 100 uM (2)
4. DCA 200 uM (2)
5. LCA 10 uM (2)
6. LCA 50 uM (2)
7. Control + DCs (3.1)
8. Sulfated DCA 100 uM (3)
9. Sulfated DCA 200 uM (3)
10. Sulfated LCA 10 uM (3)
11. Sulfated LCA 50 uM (3)
12. DCA 100 uM (3)
13. DCA 200 uM (3)
14. LCA 10 uM (3)
15. LCA 50 um (3)

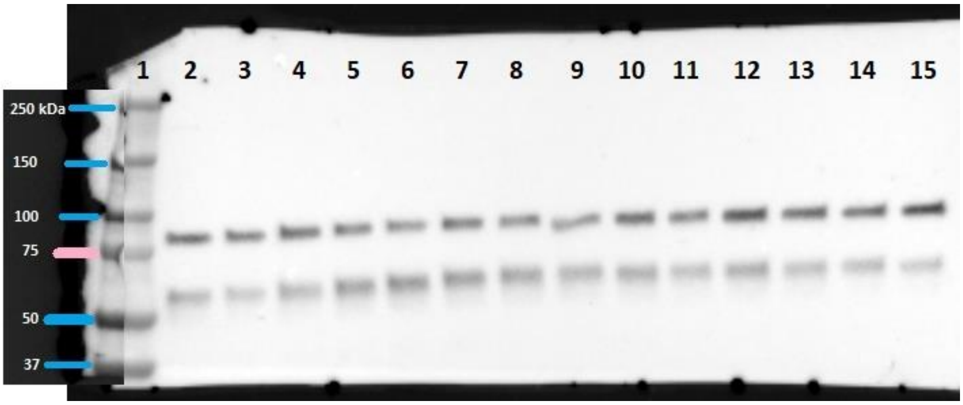

BLOT 3

1. Marker
2. Control – DCs (1.2)*
3. Control – DCs (2)
4. Control – DCs (3)
5. Control + DCs (1.2)
6. Control + DCs (2.2)
7. Control + DCs (3.2)*

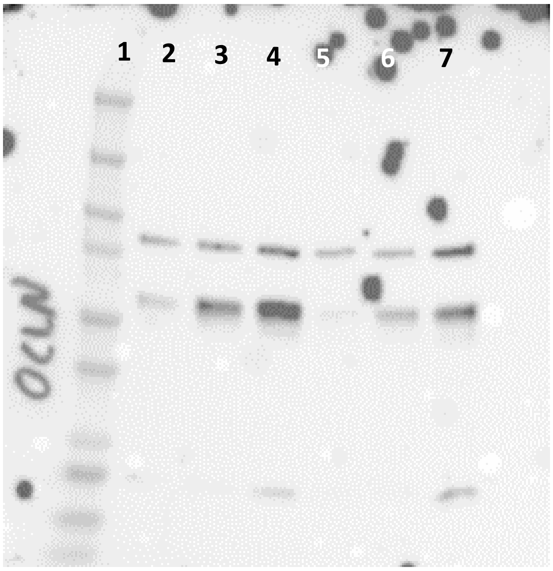

*The samples with an asterisk (*) were loaded twice in this experiment, because they were loaded on a separate gel.
N.B. The samples of BLOT 1 were used in Figure 2C.

Supplementary file 2

Zona Occludens-1 (ZO1)

BLOT 1

1. Marker
2. Control – DCs (1.1)*
3. Control + DCs (1)
4. Sulfated DCA 100 uM (1)
5. Sulfated DCA 200 uM (1)
6. Sulfated LCA 10 uM (1)
7. Sulfated LCA 50 uM (1)
8. DCA 100 uM (1)
9. DCA 200 uM (1)
10. LCA 10 uM (1)
11. LCA 50 uM (1)
12. Control + DCs (2.1)*
13. Sulfated DCA 100 uM (2)
14. Sulfated DCA (200 uM) (2)
15. Sulfated LCA 10 uM (2)

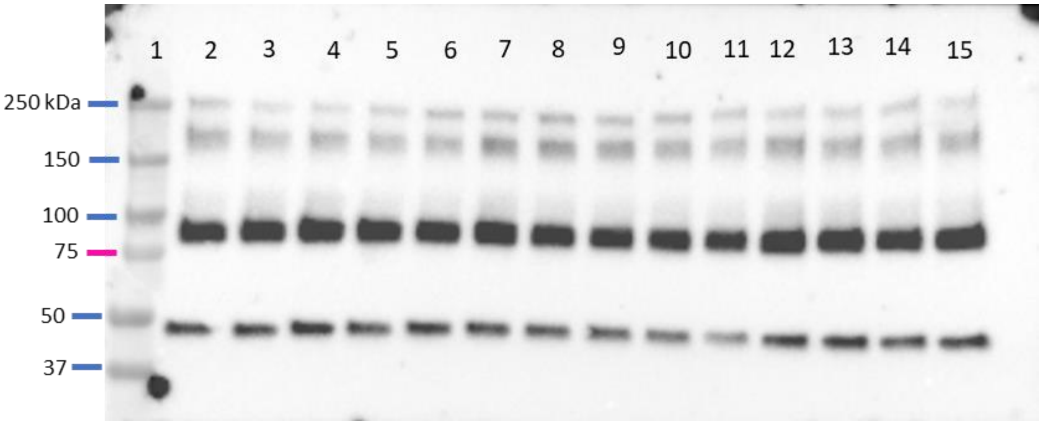

BLOT 2

1. Marker
2. Sulfated LCA 50 uM (2)
3. DCA 100 uM (2)
4. DCA 200 uM (2)
5. LCA 10 uM (2)
6. LCA 50 uM (2)
7. Control + DCs (3.1)
8. Sulfated DCA 100 uM (3)
9. Sulfated DCA 200 uM (3)
10. Sulfated LCA 10 uM (3)
11. Sulfated LCA 50 uM (3)
12. DCA 100 uM (3)
13. DCA 200 uM (3)
14. LCA 10 uM (3)
15. LCA 50 um (3)

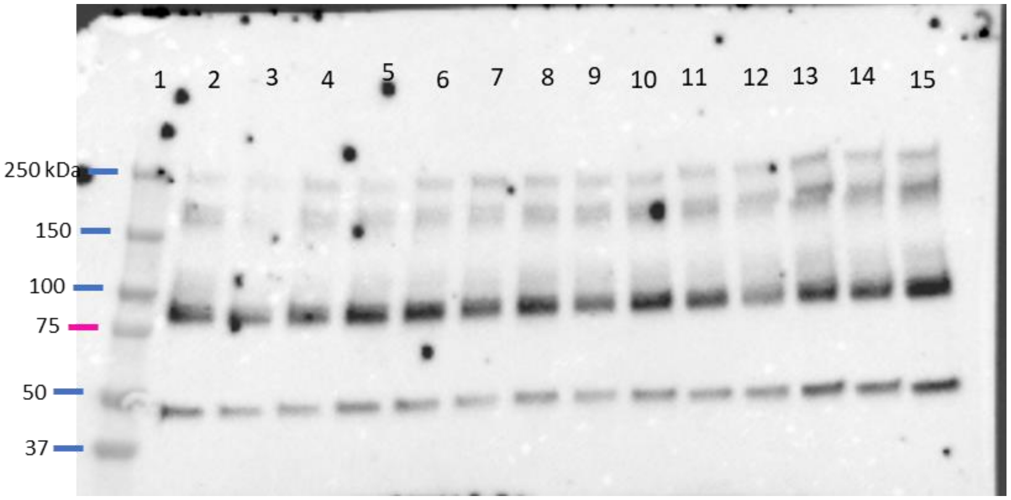

BLOT 3

1. Marker
2. Control – DCs (1.2)*
3. Control – DCs (2)
4. Control – DCs (3)
5. Control + DCs (2.2)
6. Control + DCs (3.2)*

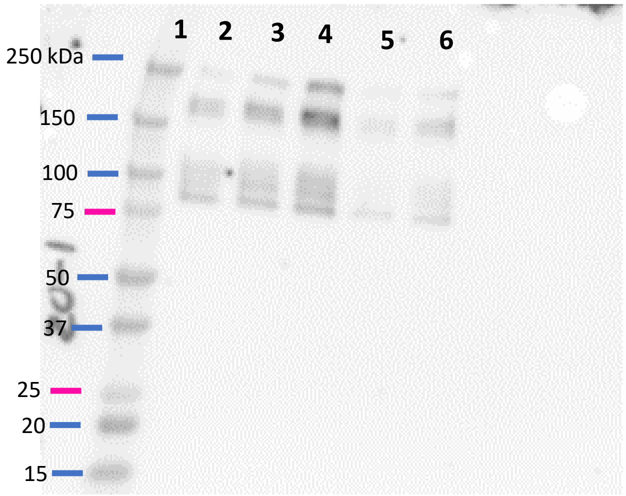

*The samples with an asterisk (*) were loaded twice in this experiment, because they were loaded on a separate gel.
N.B. The samples of BLOT 1 were used in Figure 2D.
